# Case Report: Anti-GQ1b antibody–positive overlap syndrome: a pediatric case involving the brainstem, spinal cord, and peripheral nerves

**DOI:** 10.3389/fimmu.2026.1821721

**Published:** 2026-08-06

**Authors:** Shu-Juan Zhang, Wen Zhu, Qin-Lai Ying, He-Jia Ge, Wei-Jie Yu

**Affiliations:** Department of Pediatrics, The Second Hospital of Jiaxing, Zhejiang Province, Jiaxing, China

**Keywords:** acute transverse myelitis, anti-GQ1b antibody, Bickerstaff brainstem encephalitis, case, Guillain–Barré syndrome

## Abstract

Disorders associated with anti-GQ1b antibodies predominantly encompass Bickerstaff brainstem encephalitis (BBE), Guillain–Barré syndrome (GBS), and Miller Fisher syndrome. Concurrent presentation of BBE, GBS, and acute transverse myelitis is exceptionally rare and presents significant diagnostic and therapeutic challenges. This report describes an 8-year-old boy who exhibited clinical features consistent with all three conditions, including impaired consciousness, ophthalmoplegia, ataxia, and limb weakness. Laboratory investigations revealed positive serum anti-GQ1b IgG antibodies, elevated cerebrospinal fluid protein levels with mild lymphocytic pleocytosis, and neurophysiological findings indicative of peripheral nerve demyelination. Magnetic resonance imaging of the brain and spinal cord demonstrated evidence of brainstem encephalitis and extensive spinal cord involvement. Following treatment with intravenous immunoglobulin and corticosteroid pulse therapy, marked neurological improvement was observed, and follow-up evaluations demonstrated favorable resolution of radiological abnormalities. This case highlights that anti-GQ1b antibody–associated syndrome may simultaneously affect the brainstem, spinal cord, and peripheral nerves. Early recognition and prompt initiation of combined immunotherapy may be critical for optimizing clinical outcomes.

## Introduction

Anti-GQ1b antibody syndrome encompasses a spectrum of immune-mediated disorders involving both the central nervous system (CNS) and peripheral nervous system (PNS). The condition is most commonly preceded by infectious exposures, particularly *Campylobacter jejuni* and *Mycoplasma pneumoniae*, which are associated with the induction of anti-GQ1b antibodies. These antibodies target GQ1b ganglioside antigens expressed in the oculomotor nerves and brainstem, contributing to neurological dysfunction (1, 2).

Ophthalmoplegia is a characteristic clinical manifestation of this syndrome, which includes phenotypes such as BBE, GBS, and MFS. Although traditionally regarded as distinct clinical entities, overlap presentations have been increasingly documented, with some patients exhibiting atypical features with simultaneous involvement of both CNS and PNS structures (3).

We report a rare pediatric overlap case satisfying concurrent diagnostic criteria for BBE, GBS, and ATM to enrich the phenotypic spectrum of anti-GQ1b antibody–associated disorders.

## Case report

An 8-year-old boy was admitted to the emergency department with a 5-day history of fever and altered mental status, accompanied by a 3-day history of gait instability. There was no relevant past medical history. The illness initially manifested as a low-grade fever, with a maximum tympanic temperature of 37.5°C, along with symptoms consistent with an upper respiratory tract infection. Subsequently, the clinical course progressed to include increasing mental lethargy, slowed responsiveness, gait instability, bilateral lower extremity weakness, urinary hesitancy, perioral numbness, and limb pain.

On admission, vital signs were stable. The patient was lethargic yet arousable, and demonstrated delayed responses to verbal stimuli. Neurological examination revealed impaired bilateral horizontal gaze associated with diplopia, along with left-sided peripheral facial palsy. Pathological reflexes were elicited bilaterally, including Babinski, Oppenheim, and Gordon signs. Muscle strength in both lower limbs was graded as grade IV on the Medical Research Council scale, and deep tendon reflexes of the patellar and Achilles tendons were diminished bilaterally.

Etiological investigations indicated evidence of a recent *M. pneumoniae* infection, with a positive IgM titer (2.69 COI), while testing for other infectious pathogens yielded negative results. Enzyme-linked immunosorbent assay detected serum positivity for anti-GQ1b IgG and anti-sulfatide IgG antibodies, with weak positivity for anti-GT1a antibodies and anti-GQ1b IgM antibodies. Screening for antibodies associated with autoimmune encephalitis and central nervous system demyelination was negative. Cerebrospinal fluid (CSF) examination revealed a mildly elevated leukocyte count (98 × 10^6^/L) and increased protein concentration (0.49 g/L), consistent with an atypical pleocytosis pattern. Neurophysiological assessments demonstrated multifocal peripheral nerve demyelination (**Supplementary Tables 1–4**). Needle electromyography showed no spontaneous activity with mixed recruitment patterns (**Supplementary Table 1**). Motor nerve conduction revealed reduced CMAP amplitudes in the right ulnar nerve, proximal conduction block in the right tibial nerve, and decreased F-wave persistence bilaterally (**Supplementary Table 2**). Sensory conduction was normal (**Supplementary Table 3**). Blink reflex showed absent ipsilateral R2 and contralateral R2′ responses bilaterally despite normal R1 (**Supplementary Table 4**).

**Figure 1:**
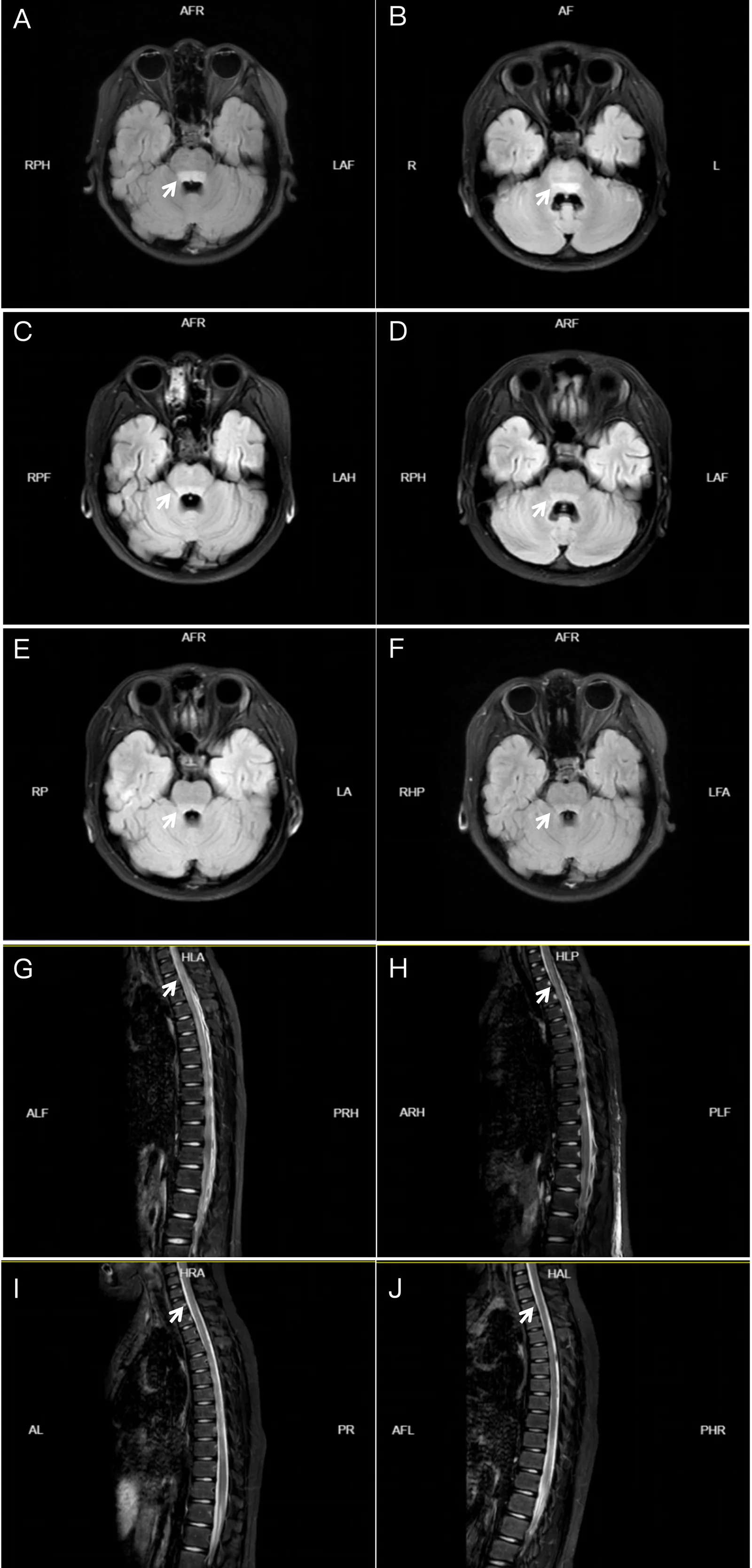
Dynamic evolution of brain and spinal cord MRI in a pediatric patient with anti-GQ1b antibody–associated overlap syndrome. **(A)** Acute-phase brain MRI (June 12, 2024; T2-FLAIR): Patchy abnormal hyperintense signals within the brainstem (arrow), consistent with acute brainstem encephalitis. **(B)** Early treatment-phase brain MRI (June 19, 2024; T2-FLAIR): Abnormal brainstem signal distribution remains comparable to that observed in the acute phase; however, signal intensity is reduced (arrow), indicating partial resolution of inflammatory edema and early therapeutic response. **(C)** Mid-treatment brain MRI (July 5, 2024; T2-FLAIR): Notable reduction in the extent of brainstem lesions is evident, compared with the acute phase; with further attenuation of signal intensity (arrow), indicating lesion absorption and ongoing treatment efficacy. **(D)** Mid-treatment brain MRI (August 10, 2024; T2-FLAIR): Continued reduction and localization of abnormal brainstem signal are evident compared with the prior examination (arrow). **(E)** Recovery-phase brain MRI (November 15, 2024; T2-FLAIR): The residual brainstem lesion remains stable in extent (arrow), with further attenuation of signal intensity, consistent with resolution of active inflammation and transition to a residual stage. **(F)** Long-term follow-up brain MRI (May 16, 2025; T2-FLAIR): Persistent but stable residual hyperintensity is noted at the posterior margin of the brainstem (arrow), compatible with gliosis or post-inflammatory structural change, with no evidence of active inflammation. **(G)** Acute-phase spinal cord MRI (June 16, 2024; sagittal STIR): Diffuse swelling of the cervical, thoracic, and lumbar spinal cord with extensive T2 hyperintensity (arrow), consistent with ATM. **(H)** Mid-treatment spinal cord MRI (June 25, 2024; sagittal STIR): Partial resolution of spinal cord swelling and reduction in abnormal signal extent compared with the acute phase (arrow). **(I)** Mid-treatment spinal cord MRI (July 14, 2024; sagittal STIR): Near-complete resolution of spinal cord swelling with marked reduction in abnormal signal intensity across all involved segments (arrow). **(J)** Recovery-phase spinal cord MRI (November 17, 2024; sagittal STIR): Restoration of near-normal spinal cord morphology with minimal residual signal (arrow), indicating substantial recovery from acute inflammatory injury.

Acute-phase brain magnetic resonance imaging (MRI) with T2-weighted fluid-attenuated inversion recovery (FLAIR) sequences revealed patchy hyperintense lesions within the brainstem, consistent with brainstem encephalitis (**Figure 1A**). Concurrent spinal MRI revealed diffuse swelling and abnormal T2 hyperintensity across the cervical, thoracic, and lumbar spinal cord segments, findings indicative of acute transverse myelitis (**Figure 1G**). One-year serial follow-up MRI confirmed gradual resolution of brainstem and spinal cord inflammatory lesions (**Figures 1B–F, H–J**). Immunological monitoring revealed a marked elevation in serum neuron-specific enolase during the acute phase (75.81 ng/mL), followed by a rapid decline after initiation of immunotherapy (18.47 ng/mL). Serum cytokine levels, including interleukin-2, interleukin-4, interleukin-6, interleukin-10, tumor necrosis factor–α, and interferon-γ, remained within reference ranges during both the acute and recovery phases.

Based on the clinical presentation and diagnostic findings, a diagnosis of anti-GQ1b antibody–associated overlap syndrome was established, comprising BBE, GBS, and ATM. Therapeutic management included intravenous immunoglobulin (IVIG; 35 g/day for 2 consecutive days, total dose 70 g) in combination with pulse therapy using methylprednisolone sodium succinate at 20 mg/kg/day for 5 days, followed by 4 mg/kg/day for 4 days, 20 mg/kg/day for 3 days, and 2 mg/kg/day for 3 days. Oral prednisone was initiated subsequently and tapered gradually over a duration of approximately 10 weeks. Concurrent anti-infective therapy and supportive symptomatic management were provided as clinically indicated.

Following initiation of immunotherapy, rapid neurological improvement was observed. By hospital day 16, the child was able to ambulate independently, with substantial improvement in ocular motor function, resolution of facial weakness, as well as normalization of reflex responses. Long-term follow-up demonstrated sustained neurological recovery with no evidence of relapse.

## Discussion

GBS comprises a heterogeneous group of immune-mediated neuropathies and is classically characterized by symmetrical limb weakness with associated hyporeflexia or areflexia (4). BBE, a recognized variant within the GBS spectrum, is clinically defined by the triad of ophthalmoplegia, ataxia, and altered consciousness (5). Anti-GQ1b antibodies represent a shared immunological marker across these related disorders and are strongly associated with specific clinical phenotypes within this spectrum. In 2014, Wakerley et al. proposed the concept of “anti-GQ1b antibody syndrome,” advocating the use of anti-GQ1b antibody positivity as a biological criterion encompassing overlapping disorders such as MFS, BBE, and acute ophthalmoplegia (6).

This report describes a rare pediatric case of anti-GQ1b antibody–positive overlap syndrome in which the clinical manifestations and laboratory findings concurrently fulfilled diagnostic criteria for BBE, GBS, and ATM, demonstrating extensive involvement across the neuroaxis. Disease phenotypes within the anti-GQ1b antibody–associated spectrum are particularly complex in pediatric populations, with substantial clinical heterogeneity and frequent overlap among syndromic entities. Previous studies have reported that approximately 39.2% of patients with anti-GQ1b antibody positivity present with overlapping MFS and GBS, 21.7% with typical GBS, and 4.8% with combined GBS and BBE phenotypes (7, 8).

Although the annual incidence of GBS is estimated at 0.4 to 1.7 cases per 100,000 individuals, reliable epidemiological data regarding anti-GQ1b antibody–associated syndromes in children remain limited (9). Review of the available literature indicates that although overlap syndromes involving GBS and BBE have been reported, confirmed involvement of the spinal cord parenchyma remains exceptionally rare (10, 11). Odaka et al. systematically characterized the GBS–BBE overlap syndrome, identifying clinical features such as impaired consciousness, ataxia, and reduced or absent tendon reflexes, and demonstrated the presence of serum anti-GQ1b antibodies in the majority of affected patients (12). In a cohort study of 68 pediatric patients with anti-GQ1b antibody–associated syndromes, abnormal intracranial magnetic resonance imaging findings were reported in only 18% of cases, with no observed evidence of spinal cord parenchymal involvement on imaging or neurophysiological evaluation (13). Similarly, a retrospective analysis of 18 pediatric patients with BBE reported mild spinal cord signal abnormalities in only three cases (approximately 16.7%), with lesions confined to the upper cervical segments (C1–C2) and without extension to thoracic or lumbar levels (14). In contrast, the present case demonstrated typical brainstem lesions and peripheral nerve demyelination in conjunction with extensive involvement of the cervical, thoracic, and lumbar spinal cord segments. This multisite involvement is likely related to the widespread distribution of GQ1b ganglioside antigens throughout the CNS and PNS. The findings in this case expand the known clinical spectrum of anti-GQ1b antibody–associated disorders and indicate that, in pediatric patients, the condition may manifest as a diffuse overlap syndrome involving multiple levels of the nervous system. In this case, the cerebrospinal fluid showed mild pleocytosis (98 × 10^6^/L) with mildly elevated protein (0.49 g/L), which is inconsistent with the characteristic albuminocytological dissociation of typical Guillain-Barré syndrome (GBS) but rather aligns with acute inflammatory changes of the central nervous system. This finding is consistent with the imaging and clinical phenotypes of spinal cord parenchymal involvement in the patient, suggesting that the cerebrospinal fluid inflammatory response may have originated from spinal cord injury and increased blood-brain barrier permeability.

This pediatric patient tested positive for multiple antiganglioside antibodies, a clinically important serological finding. Serum testing revealed positive anti-GQ1b IgG and anti-sulfatide IgG antibodies, with weakly positive anti-GT1a IgG antibodies. The coexistence of multiple antiganglioside antibodies may produce synergistic effects through distinct immunopathological pathways, exacerbating immune-mediated neural injury. Suzuki et al. reported a case with multiple antiganglioside antibody positivity presenting with multiple cranial nerve palsies and extensive cranial nerve enhancement on MRI (15). Gómez-Dabó et al. further described a rare variant of Guillain-Barré syndrome with multi-ganglioside reactivity, in which the patient developed severe cranial nerve involvement attributable to the combined effects of multiple antibodies, confirming the clinical relevance of multi-antibody positivity with extensive neurological involvement (16). In the present case, anti-sulfatide antibodies primarily contributed to peripheral nerve demyelination and could exacerbate axonal and myelin damage through complement activation, consistent with the observed lower extremity weakness; anti-GQ1b and anti-GT1a antibodies, which exhibit immunological cross-reactivity, jointly participated in the pathogenesis of ophthalmoplegia and brainstem involvement. The synergistic pathogenic effects of these multiple antibodies provide a reasonable explanation for the complex clinical phenotype characterized by severe limb weakness, ophthalmoplegia, and brainstem dysfunction observed in this patient, thereby expanding the recognized immunological spectrum of anti-GQ1b antibody-associated syndromes. Clinicians should remain vigilant for more extensive and severe neurological injury in pediatric patients with multiple antiganglioside antibody positivity, and comprehensive neuroaxis evaluation with early intervention should be promptly initiated.

Central nervous system involvement in anti-GQ1b antibody syndrome is typically restricted to the brainstem, and no definitive evidence supports the notion that these antibodies can directly mediate spinal cord parenchymal damage. The extensive spinal cord involvement observed in this patient, affecting the cervical, thoracic, and lumbar segments, may be explained by the “double-hit” hypothesis—that is, the coexistence of anti-GQ1b antibody syndrome and an independent post-infectious acute transverse myelitis. Mycoplasma pneumoniae infection can induce anti-GQ1b antibody production through molecular mimicry while triggering localized immune responses within the spinal cord, thereby creating a superimposed condition of anti-GQ1b syndrome and post-infectious myelitis. Additionally, low-affinity cross-reactivity between anti-GQ1b antibodies and spinal gangliosides cannot be excluded. The cerebrospinal fluid findings of mild pleocytosis with elevated protein support the presence of an independent inflammatory process within the spinal cord. The spinal cord involvement in this case may represent a rare expanded phenotypic spectrum of the disease; however, the underlying immunological mechanisms warrant further investigation in larger cohorts.

The differential diagnosis requires careful prioritization based on neuroaxis involvement patterns. Encephalomyeloradiculoneuropathy (EMRN) and anti-GQ1b antibody–associated overlap syndrome both feature combined central and peripheral nervous system lesions, distinguishing them from disorders confined to a single neuroanatomical compartment. EMRN is characterized by serum and CSF anti-neutral glycolipid antibodies, including anti-lactosylceramide, anti-glucosylceramide, and anti-galactosylceramide, markers atypical of anti-GQ1b antibody syndrome (17, 18). Both conditions may present with extensive long-segment spinal cord involvement; however, this patient demonstrated the classic BBE triad, seropositivity for anti-GQ1b, anti-GT1a, and anti-sulfatide antibodies, and showed no cerebral white matter lesions, basal ganglia involvement, or spinal nerve root enhancement—findings frequently observed in EMRN. Documented preceding Mycoplasma pneumoniae infection and rapid neurological recovery after combined immunotherapy further support anti-GQ1b overlap syndrome. EMRN predominantly affects adults, with pediatric cases being exceedingly rare. All serum and CSF clinical specimens were depleted, precluding anti-neutral glycolipid antibody profiling and leaving residual uncertainty regarding complete EMRN exclusion.

Secondary differential diagnoses include ADEM, NMOSD, and multiple sclerosis. These disorders are largely confined to the CNS, unlike this patient’s widespread combined neuroaxial injury. ADEM typically presents with discontinuous short-segment spinal lesions and multifocal cerebral white matter changes, none of which were observed in this patient, who instead showed continuous C2–L3 involvement with homogeneous signal. Negative serum AQP4-IgG and MOG-IgG excluded NMOSD, and the lack of spatially separated inflammatory lesions failed to meet the McDonald criteria for multiple sclerosis.

In this case, peripheral blood cytokine concentrations remained within reference ranges, whereas the CSF analysis demonstrated marked lymphocytic pleocytosis (19). This pattern, reflecting relative peripheral immune quiescence in the presence of pronounced CNS inflammation, supports a predominantly antibody-mediated pathogenic mechanism, with immune injury localized to neural tissues. In addition, the presence of anti-sulfatide antibodies may have contributed to increased disease severity, as coexisting antiganglioside antibodies have been reported to exert synergistic effects that exacerbate immune-mediated neural damage (20).

These findings offer further insight into the immunopathogenesis of this disorder. In pediatric populations, immaturity of the blood–brain barrier may facilitate the translocation of circulating autoantibodies into the CNS, promoting localized inflammatory responses (21). This mechanism may partly account for the propensity for the greater extent of CNS involvement observed in children compared with adults, in whom lesion distribution tends to be more focal or limited.

Approximately two-thirds of patients with anti-GQ1b antibody–associated syndromes report a preceding infectious illness. In the present case, antecedent *M. pneumoniae* infection was identified. The surface lipopolysaccharide structures of this pathogen share molecular similarities with GQ1b ganglioside components expressed on neural tissues (22–24). Such molecular mimicry may trigger the production of cross-reactive antibodies that target host neural antigens, highlighting the contributory role of infectious environmental factors in disease initiation and progression.

In pediatric patients presenting with acute muscle weakness accompanied by ophthalmoplegia or altered mental status, prompt assessment of serum anti-GQ1b antibodies and whole-spine magnetic resonance imaging may be warranted as part of the initial diagnostic evaluation (25). Implementation of this approach in the present case enabled establishment of a definitive diagnosis within four days of hospital admission, substantially reducing the diagnostic interval.

For severe cases of ATM, current clinical guidelines recommend high-dose corticosteroids—typically intravenous methylprednisolone, administered alone or in combination with intravenous immunoglobulin (IVIG) or plasma exchange (26). IVIG has demonstrated therapeutic benefit in the treatment of GBS; however, evidence supporting its use in BBE and other anti-GQ1b antibody–associated disorders remains limited (27, 28). In this case, early initiation of combination therapy with IVIG and high-dose methylprednisolone pulse therapy, followed by gradual tapering with oral corticosteroids, was undertaken. This therapeutic strategy is intended to reduce circulating autoantibody levels while concurrently suppressing inflammatory activity within the CNS (29–31). Follow-up assessments demonstrated favorable clinical outcomes, and serial magnetic resonance imaging confirmed progressive resolution of lesions within the brainstem and spinal cord. Serum concentrations of neuron-specific enolase declined markedly from 75.81 ng/mL to 18.47 ng/mL; notably, normalization of neuron-specific enolase levels preceded the recovery of muscle strength by approximately one week, indicating potential utility of this biomarker for early monitoring of treatment response (32, 33).

This study has limitations. Retrospective design and depleted stored clinical specimens precluded anti-neutral glycolipid antibody testing, limiting complete EMRN exclusion. Future prospective cohorts with sample banking should adopt this panel. As a single pediatric case, larger multi-center studies are needed to clarify whether extensive spinal lesions represent an expanded anti-GQ1b phenotype or concurrent post-infectious myelitis.

## Conclusion

This case demonstrates that anti-GQ1b antibody syndrome may present as a severe overlapping syndrome affecting the brainstem, peripheral nerves, and spinal cord. Notably, this patient exhibited extensive spinal cord involvement, particularly in the cervical, thoracic, and lumbar segments. The etiology remains unclear and may represent either an atypical manifestation of anti-GQ1b-mediated disease or a coexisting independent process, such as post-infectious myelitis. After systematic differential diagnosis prioritizing encephalomyeloradiculoneuropathy alongside acute disseminated encephalomyelitis, neuromyelitis optica spectrum disorder, and multiple sclerosis, and excluding the latter three, integration of clinical, serological, neurophysiological, and neuroimaging findings broadens the recognized phenotypic spectrum of the disease and highlights a distinctive immunopathological profile in pediatric patients, characterized by relative peripheral immune quiescence in the presence of active CNS inflammation. These observations contribute to improved understanding of antibody-mediated autoimmune mechanisms targeting neural tissues and delineate clinical and neuroimaging features associated with widespread CNS involvement. The findings further emphasize the value of prompt diagnostic assessment and highlight the potential therapeutic benefit of early combined immunotherapeutic strategies in appropriate clinical settings.

## Data Availability

The datasets used and/or analyzed during the current study are available from the corresponding author upon reasonable request.
