# Supplementary material for: Case Report: Anti-GQ1b antibody–positive overlap syndrome: a pediatric case involving the brainstem, spinal cord, and peripheral nerves
Source: Front Immunol. 2026 Aug 6;17:1821721. doi: 10.3389/fimmu.2026.1821721 (PMC13490084; doi:10.3389/fimmu.2026.1821721)
Supplement: Supplementary file 1 [file DataSheet1.docx]

Table S1: Electromyography results

| Tested muscle | Side | Rest activity | Motor unit potentials at mild contraction | Recruitment pattern at maximal contraction | Conclusion |
| --- | --- | --- | --- | --- | --- |
| Biceps brachii | L | No spontaneous discharges | Not recordable | Mixed pattern | Normal |
| Extensor indicis | R | No spontaneous discharges | Not recordable | Mixed pattern | Normal |
| Deltoid | R | No spontaneous discharges | Not recordable | Mixed pattern | Normal |
| Rectus femoris | L | No spontaneous discharges | Not recordable | Mixed pattern | Normal |
| Vastus medialis | R | No spontaneous discharges | Not recordable | Mixed pattern | Normal |
| Tibialis anterior | R | No spontaneous discharges | Not recordable | Mildly reduced mixed pattern | Normal |

Note: At rest, no spontaneous potentials such as fibrillation potentials or positive sharp waves were observed in any of the examined muscles. During strong contraction, the right tibialis anterior muscle exhibited a simple mixed phase, while the remaining examined muscles showed a mixed phase, indicating no definitive signs of denervation or neurogenic damage within the examined range. This finding is inconsistent with axonal damage and supports the predominance of demyelinating pathology.

Table S2 Motor Nerve Conduction Studies of Upper and Lower Limbs

| Tested Nerve | Stimulation – Recording Site | Latency (ms) | Amplitude (mV) | Conduction Velocity (m/s) | F-wave (ms) | Abnormality |
| --- | --- | --- | --- | --- | --- | --- |
| Left ulnar nerve (ADM recording) | Wrist–ADM | 1.82 | 5.3 | – | 22.5 | Normal |
|  | Elbow–wrist | 5.19 | 4.9 | 51.9 | – | Normal |
|  | Distal elbow–elbow | 4.3 | 4.7 | – | – | Normal |
|  | Proximal elbow–distal elbow | 5.92 | 4.4 | 61.7 | – | Normal |
|  | Mid-arm–proximal elbow | 6.73 | 4.3 | 61.7 | – | Normal |
| Right ulnar nerve (ADM recording) | Wrist–ADM | 1.83 | 2.9 | – | 22.7 | Reduced CMAP amplitude |
|  | Elbow–wrist | 5.13 | 3.2 | 51.5 | – | Reduced CMAP amplitude |
|  | Distal elbow–elbow | 4.03 | 2.7 | – | – | Reduced CMAP amplitude |
|  | Proximal elbow–distal elbow | 5.84 | 2.9 | 55.2 | – | Reduced CMAP amplitude |
|  | Mid-arm–proximal elbow | 7.15 | 2.8 | 61.1 | – | Reduced CMAP amplitude |
| Left median nerve (motor) | – | – | – | – | 22.2 | Decreased F-wave persistence |
| Right median nerve (motor) | Wrist–APB | 2.67 | 7.3 | – | 22.9 | Decreased F-wave persistence |
|  | Elbow–wrist | 5.71 | 7 | 54.3 | – | Normal |
|  | Axilla–elbow | 6.86 | 6.8 | 47.8 | – | Normal |
| Left tibial nerve (motor) | Ankle–AH | 2.19 | 5.3 | – | 37.9 | Decreased F-wave persistence |
|  | Popliteal fossa–ankle | 10.2 | 6.5 | 44.9 | – | Normal |
| Right tibial nerve (motor) | Ankle–AH | 3.48 | 6.9 | – | 38.5 | Decreased F-wave persistence |
|  | Popliteal fossa–ankle | 11.2 | 3.2 | 42.1 | – | Proximal conduction block |
| Left common peroneal nerve (motor) | Ankle–EDB | 4.13 | 3.6 | – | – | Normal |
|  | Fibular head–ankle | 9.94 | 3.7 | 48.2 | – | Normal |
| Right common peroneal nerve (motor) | Ankle–EDB | 2.8 | 4 | – | – | Normal |
|  | Fibular head–ankle | 9.83 | 3.3 | 43.4 | – | Normal |

Note: Sensory-motor dissociation was observed in motor nerve conduction. Amplitude reduction >50% at the popliteal segment of the right tibial nerve suggests proximal mixed nerve lesion. Diffusely reduced amplitudes of the right ulnar nerve indicate mild axonal involvement. Reduced F-wave persistence bilaterally implies radicular irritation. The remaining motor nerve conduction parameters are basically normal.

ADM: Abductor digiti minimi, APB: Abductor pollicis brevis, AH: Abductor hallucis, EDB: Extensor digitorum brevis, CMAP: Compound muscle action potential.

Table S3 Sensory Nerve Conduction of Upper and Lower Limbs

| Tested Nerve | Stimulation–Recording Site | Latency (ms) | Amplitude (μV) | Conduction Velocity (m/s) | F-wave (ms) | Findings |
| --- | --- | --- | --- | --- | --- | --- |
| Left ulnar sensory | Digit V – Wrist | 2.17 | 12.7 | 59.2 | – | Normal |
| Right ulnar sensory | Digit V – Wrist | 2.18 | 11.1 | 49.7 | – | Normal |
| Right median sensory | Digit I – Wrist | 1.77 | 24.6 | 55.1 | – | Normal |
|  | Digit II – Wrist | 2.23 | 19.8 | 57 | – | Normal |
|  | Digit III – Wrist | 2.19 | 16.1 | 55.2 | – | Normal |
| Left superficial peroneal sensory | Lateral calf – Dorsum of foot | 2.6 | 11.5 | 47.9 | – | Normal |
| Right superficial peroneal sensory | Lateral calf – Dorsum of foot | 2.95 | 9.3 | 49.8 | – | Normal |
| Left sural sensory | Posterolateral calf – Lateral malleolus | 2.85 | 21.4 | 50 | – | Normal |
| Right sural sensory | Posterolateral calf – Lateral malleolus | 3.15 | 15.6 | 51.1 | – | Normal |

Note: Amplitude and conduction velocity of all tested upper and lower limb sensory nerves (median, ulnar, superficial peroneal and sural nerves) remain within normal limits. Combined with abnormal motor nerve findings in Table 2, the electrophysiological features are consistent with predominantly motor polyneuropathy (variant of GBS).

Table S4 Blink Reflex Results

| Stimulation Site | Recording Side | R1 Latency (ms) | R1 RefDev | R2 Latency (ms) | R2 RefDev | Interpretation |
| --- | --- | --- | --- | --- | --- | --- |
| Left supraorbital nerve | Ipsilateral (Left) | 9.4 | Within normal range | Not elicited | – | Normal R1; absent R2 |
| Left supraorbital nerve | Contralateral (Right) | – | – | Not elicited | – | Absent contralateral R2′ |
| Right supraorbital nerve | Ipsilateral (Right) | 10.1 | Within normal range | Not elicited | – | Normal R1; absent R2 |
| Right supraorbital nerve | Contralateral (Left) | – | – | Not elicited | – | Absent contralateral R2′ |
| Bilateral R1 latency difference | – | −0.67 | Normal | – | – | Within normal limits |

Note: R1 responses are bilaterally normal, whereas ipsilateral R2 and contralateral R2′ cannot be elicited, indicating abnormal blink reflex. RefDev=Reference Deviation
